# Role of Caveolae family-related proteins in the development of breast cancer

**DOI:** 10.3389/fmolb.2023.1242426

**Published:** 2023-09-27

**Authors:** Qinyu Han, Shi Qiu, Huiwen Hu, Wenjing Li, Xiangqi Li

**Affiliations:** ^1^ Department of Breast Center, The Second Affiliated Hospital of Shandong First Medical University, Tai’an, Shandong, Chinaa; ^2^ Department of the First Clinical Medical School, Shandong University of Traditional Chinese Medicine, Jinan, Shandong, China

**Keywords:** breast cancer, Caveolae, Caveolins, Cavins, targeted therapy

## Abstract

Breast cancer has become the most significant malignant tumor threatening women’s lives. Caveolae are concave pits formed by invagination of the plasma membrane that participate in many biological functions of the cell membrane, such as endocytosis, cell membrane assembly, and signal transduction. In recent years, Caveolae family-related proteins have been found to be closely related to the occurrence and development of breast cancer. The proteins associated with the Caveolae family-related include Caveolin (Cav) and Cavins. The Cav proteins include Cav-1, Cav-2 and Cav-3, among which Cav-1 has attracted the most attention as a tumor suppressor and promoting factor affecting the proliferation, apoptosis, migration, invasion and metastasis of breast cancer cells. Cav-2 also has dual functions of inhibiting and promoting cancer and can be expressed in combination with Cav-1 or play a regulatory role alone. Cav-3 has been less studied in breast cancer, and the loss of its expression can form an antitumor microenvironment. Cavins include Cavin-1, Cavin-2, Cavin-3 and Cavin-4. Cavin-1 inhibits Cav-1-induced cell membrane tubule formation, and its specific role in breast cancer remains controversial. Cavin-2 acts as a breast cancer suppressor, inhibiting breast cancer progression by blocking the transforming growth factor (TGF-β) signaling pathway. Cavin-3 plays an anticancer role in breast cancer, but its specific mechanism of action is still unclear. The relationship between Cavin-4 and breast cancer is unclear. In this paper, the role of Caveolae family-related proteins in the occurrence and development of breast cancer and their related mechanisms are discussed in detail to provide evidence supporting the further study of Caveolae family-related proteins as potential targets for the diagnosis and treatment of breast cancer.

## 1 Introduction

Breast cancer (BC) is one of the most common malignant tumors in women worldwide. In recent years, the incidence of breast cancer has increased significantly, and it shows a trend of younger age, which not only causes serious harm to women’s health and quality of life but also seriously increases the resulting social and economic burden (Heer et al., 2020). At present, despite remarkable progress in targeted therapy, hormone therapy, surgery, chemotherapy and radiotherapy, breast cancer is still the main cause of cancer death in women (Waks et al., 2019; Sung et al., 2021). The occurrence and development of breast cancer is a multistep process, and the expression of Caveolae family-related proteins is closely related to the proliferation, apoptosis, migration, invasion, metastasis and drug resistance of breast cancer cells, playing a dual role in inhibiting and promoting cancer.

A Caveola is formed by the inward depression of the plasma membrane of the cell. It is a special cystic structure rich in cholesterol and forms a “flask” structure with a diameter of 60–80 nm on the cell membrane, also known as cell membrane Cave-like invagination. Its main function is to regulate certain signaling molecules. As a signal transduction hub, it is involved in transmembrane material transport and various intercellular interactions (Filippini and D'Alessio, 2020). It has always been believed that the function of Caveolae is mainly to participate in transmembrane material transport. Recent studies have found that Caveolae are also hubs of cell signaling molecule enrichment and signal transduction and play a direct regulatory role in the activity of many key signaling molecules. The integrity of the Caveolae is closely related to tumor cell function (Parton Caveolae, 2018; Singh and Lamaze, 2020). Caveolae, as cytoplasmic membrane structures, are mainly composed of lipids and proteins. The lipid components mainly include cholesterol, glycosphingolipids (GSLs) and sphingomyelin (SPH), which constitute the lipid core of Caveolae. If the cholesterol content of Caveolae is reduced too much, the number of Caveolae invaginations will be reduced. Similarly, blocking cholesterol transport to the cell surface pharmacologically indicates that cholesterol plays an important role in maintaining the structure of Caveolae (Fernandes and Oliveira-Brett, 2020).

Caveolae family-related proteins, also known as Caveolins and Cavins, are the main components of the Caveolae and play important roles in various physiological and pathological processes, such as cell endocytosis, maintenance of lipid homeostasis, signal transduction and the occurrence and development of tumors (Part et al., 2020). Mammalian Caveolins (Cavs) include Cav-1, Cav-2, and Cav-3. Cavins include Cavin-1 (polymerase 1 transcription release factor (PTRF)), Cavin-2 (serum deprivation response factor (SDPR)), Cavin-3 (c kinase binding SDR-associated gene product (SRBC)) and Cavin-4 (muscle-restricted friable protein (MURC)) (Parton et al., 2018). Because of the differences in the structure and function of these proteins, they have different biological effects in the body. However, the specific mechanism of action and the exact function are still unclear, and there are still many controversies. In this paper, the role of Caveolae family-related proteins in breast cancer is summarized as follows.

## 2 Relationship between Caveolins and breast cancer

### 2.1 Cav-1 and breast cancer

**FIGURE 1:**
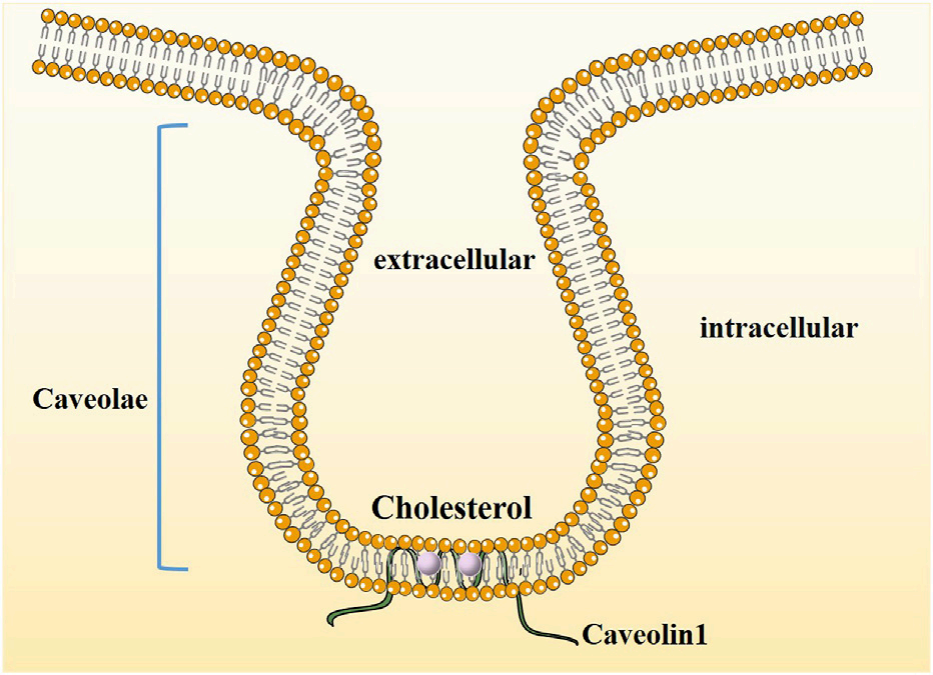
The structure of Caveolin1. Caveolae are cholesterol-enriched, rigid membrane microdomains that are composed of scaffold proteins named caveolins. The most important constituent protein is Caveolin-1.

Cav-1, a member of the Caveolae family-related proteins was discovered and reported by Palade in 1953 when he observed mouse capillary endothelial cells (Palade, 1953) and was identified and cloned by Rothberg et al. (Rothberg et al., 1992) in 1992. Cav-1, an integrated membrane protein composed of 178 amino acid residues with a molecular weight of 22 kDa, is one of the main scaffold proteins of Caveolae (Figure 1). Its expression is missing in breast cancer, lung cancer, ovarian cancer and osteosarcoma and may be related to its abnormal promoter methylation (Parat and Riggins, 2012).

Sgara et al. (Sagara et al., 2004) found that Cav-1 can regulate the distribution and translocation of ER in normal breast epithelium and is closely related to progesterone receptors to maintain normal breast development. At present, there are two views on the correlation between Cav-1 and breast cancer: 1) Cav-1 acts as a suppressor gene of breast cancer, which inhibits the malignant process of breast cancer; 2) Cav-1 expression promotes the occurrence and development of breast cancer. Some researchers support the first view because low or absent levels of Cav-1 mRNA and protein can be seen in tumor tissue samples from human primary breast cancer patients, oncogene-transformed cells, transgenic mouse breast cancer models, and some human or mouse transformed epithelial cell lines (Razani et al., 2000). Later, some scholars designed experiments to force the re-expression of Cav-1 in transformed breast cancer cells, which not only eliminated the cancerous potential of these cells but also inhibited aggressiveness (Zhang et al., 2000; Fiucci et al., 2002). Other researchers support the second view, and their evidence is that the Cav-1 gene locus is located at the aggregation point of suppressor genes in many human epithelial origin malignancies, including breast cancer (Zen et al., 1995; Zenklusen et al., 1995). However, until now, there has been no unified understanding of the mechanism of the association between Cav-1 and breast cancer.

#### 2.1.1 Effect of Cav-1 on the proliferation of breast cancer cells

At present, the findings of most studies suggest that Cav-1 can inhibit the proliferation of breast cancer and act as a tumor suppressor in the occurrence and development of breast cancer (Qian et al., 2019). Research results have shown that compared with normal breast tissue and adjacent tissues, the expression of Cav-1 protein in breast cancer tissue is low, and inhibiting the expression of Cav-1 by gene knockout in normal breast epithelial cells can significantly upregulate the expression of related tumor growth factors (Shi et al., 2016). For example, stromal cell derived factor-1 (SDF-1), epidermal growth factor (EGF), and fibroblast specific protein-1 (FSP-1) promote the growth and carcinogenesis of mammary epithelial cells (Shi et al., 2016).

In addition, Cav-1 can also affect the proliferation of breast cancer by inhibiting aerobic glycolysis in breast cancer (Warburg effect) (Jiao et al., 2019). Aerobic glycolysis is the most common form of energy recombination in tumors. Even in the presence of sufficient oxygen, tumor cells still rapidly supply energy through glycolysis to meet their abnormally high proliferation and metabolic energy needs. Mechanistically, Cav-1 reduces the entry of p65/p50 into the nucleus by inhibiting the phosphorylation of NF-κB, thus inhibiting the transcriptional activation of C-myc, which, as a classical aerobic glycolysis-related protein, mediates the orderly progression of aerobic glycolysis. Therefore, high expression of Cav-1 can lead to a decrease in glucose in the cell and in the production of lactic acid and to low expression of lactate dehydrogenase A (LDH-A) and 3-phosphoinositol-dependent protein kinase-1 (PDK1), the key enzymes in glycolysis, thus inhibiting aerobic glycolysis and hence the proliferation of breast cancer cells. In recent years, the role of Cav-1 in negatively regulating the proliferation of breast cancer by inhibiting autophagy has been widely studied. Cav-1 inhibits the production of autophagic lysosomes by blocking the fusion of lysosomes and autophagosomes, thus blocking autophagic flow and inhibiting the proliferation of breast cancer cells (Shi et al., 2015).

The inactivation of the Cav-1 gene leads to increased (Estrogen Receptor alpha) ERα expression, which can be seen in both human breast cancer epithelial cells and mouse primary breast cancer epithelial cells, and can promote the growth of stimulated three-dimensional epithelioid structures (Li et al., 2006). Earlier studies have shown that downregulation of Cav-1 can stimulate the expression of ERα, which also indicates that Cav-1 may play an important regulatory role in the maintenance of normal cell growth in breast tissue (Zhang et al., 2005). Therefore, mutation or low expression of Cav-1 is undoubtedly a factor promoting the progression of ERα (+) breast cancer. It is a risk factor for a poor breast cancer prognosis.

Other studies have shown that downregulation of Cav-1 in breast cancer MCF-7 cells can increase the expression of large conductive Ca^2+^-activated potassium channels (BKCa) in the cell membrane and promote cell proliferation (Du et al., 2014). Cav-1 can also promote the binding of epidermal growth factor receptor (EGFR) to the kinase domain of the Caveolin binding motif and act as a pro-cancer factor to activate EGFR-mediated mitosis initiation (Liang et al., 2018). Studies have shown that abnormal expression of Cav-1 can enhance EGFR signaling and increase the malignant potential of MCF-7 breast cancer cells, while overexpression of Cav-1 can enhance the cell growth inhibition ability of EGFR tyrosine kinase inhibitors (Agelaki et al., 2009).

Cav-1 negatively regulates cell proliferation by inhibiting the expression of cyclin D1. Overexpression of Cav-1 can induce upregulation of the cell cycle Factors p21, p27 and cyclin B1 and downregulation of cyclin D2, which leads to G2/M phase arrest of cells. Inhibition of proliferation of breast cancer MDA-MB-231 and MCF-7 cells (Kang et al., 2016). In addition, under normal circumstances, Cav-1 can inhibit the Cyclin Dl promoter and downregulate the expression of Cyclin D1, thereby blocking cells in the G0/G1 phase and regulating the cell cycle. In addition, in breast cancer tumor tissues of ERa (+) breast cancer patients, Cyclin D1 overexpression was also observed in samples with Cav-1 mutations. ERa was not expressed in tissues negative for Cyclin D1 immunostaining, and Cav-1 gene expression was normal (Li et al., 2006). Therefore, the inactivation of Cav-1 is likely to occur in the early stage of breast transformation and induce increased sensitivity to estrogen, upregulating ERa, and simultaneously to induce upregulation of Cyclin D1 expression. In other words, the amplification or overexpression of Cyclin D1 and ERa in breast cancer may occur through the same pathway. Both are induced by mutations in Cav-1.

Studies have shown that the interstitium of breast cancer cells is usually in a high-oxygen microenvironment, and the transport efficiency of Cav-1 into cells is low (Monti et al., 2017). In the hypoxic TME, bisphenol A (BPA) in cells can induce the competitive binding of G protein estrogen receptor (GPER) to Cav-1, resulting in the release of heat shock protein 90 (HSP90), activation of hypoxia-inducing factor-1α (HIF-1α) and vascular endothelial growth factor (VEGF), and promotion of cell proliferation (Xu et al., 2017).

It is concluded that Cav-1 can affect cell proliferation in breast cancer by inducing changes in receptor activity and membrane surface ion channels, regulating the cell cycle, and regulating the tumor microenvironment (TME) and intercellular interactions.

#### 2.1.2 Effect of Cav-1 on apoptosis of breast cancer cells

At present, the role of Cav-1 in breast cancer apoptosis is still controversial, and there are different studies on its ability to promote or inhibit apoptosis (Wang et al., 2014a; Chen et al., 2019). Regarding the promotion of Cav-1 apoptosis in breast cancer cells, studies have found that docetaxel (DTX) can upregulate Cav-1 in MCF-7 and MDA-MB-231 breast cancer cells, thereby regulating apoptosis pathway-related proteins, Bcl-2 phosphorylation, p53 and Bax expression, and cleaved PARP cleavage (Kang et al., 2016). In addition, Shi et al. (2016) found that the expression of P53 and apoptosis regulator (TIGAR) was upregulated in BT474 cells, and apoptosis was thus inhibited. Subsequently, Shi et al. (2015) found that Cav-1 deletion and lipid raft breakdown could increase V-ATPase activity, activate autophagy‒lysosome fusion, improve autophagy levels and inhibit apoptosis.

However, Wang et al. (2014b) found that in breast ductal cancer cells (BT474), Cav-1 can activate the extracellular signal-regulating kinase ERK1/2 signaling pathway, upregulate the expression of cell cyclin D1 and β-catenin-related factors, reduce G0/G1 phase arrest and increase the proportion of S-phase cells. At the same time, the expression of the autophagy-related proteins Beclin-1, light chain 3-Ⅱ and Atg12/5 was enhanced, which promoted the formation of autophagosomes and inhibited apoptosis. Cav-1 knockdown inhibited cell migration and invasion. Badana et al. (2018) also proposed that methyl-β-cyclodextrin (MβCD) in lipids can downregulate the expression of Cav-1 and Wnt receptor LRP6, synergistically affect the expression of survivin, Bcl-2 and Bax, induce the breakdown of lipid rafts, and mediate apoptosis.

At present, the effect of Cav-1 on the apoptosis of breast cancer cells is mainly dependent on apoptosis-related proteins and autophagy. However, there is no consensus on whether Cav-1 promotes or inhibits autophagy, which may be related to the stage of Cav-1’s influence on autophagy.

#### 2.1.3 Effect of Cav-1 on the invasion and metastasis of breast cancer

Invasion and metastasis of breast cancer are among the important characteristics of malignant progression of breast cancer and represent the main challenge in the clinical prevention and treatment of breast cancer. Studies have shown that the expression of Cav-1 protein in MDA-MB-231 cells of metastatic breast cancer is significantly higher than that in MCF-7 cells of nonmetastatic breast cancer, and there is also a positive correlation between metastasis ability and Cav-1 protein expression in breast cancer tissues (Alevizos et al., 2014). Current research on breast cancer metastasis mainly focuses on epithelial-mesenchymal transformation (EMT), extracellular matrix (ECM) changes, cytoskeleton reconstruction, and angiogenesis (Lu and Kang, 2019). Interestingly, Cav-1 plays an important role in these processes and can affect the migration and invasion of breast cancer cells by regulating the expression of epithelial mesenchymal transition (EMT)-related markers, matrix metalloproteinases (MMPs) and Rho-GTPases. It can affect the metastasis of breast cancer by regulating the expression of metastasis-related proteins and apoptosis of cells.

It has been reported that the decreased expression of Cav-1 affects the expression of EMT-related genes, such as the increased expression of E-cadherin, while the decreased expression of EMT-related transcription factors such as Vimentin, Snail and Slug can inhibit the occurrence of EMT. In addition, during high sugar-induced EMT, it has been observed that inhibition of the estrogen receptor signaling pathway leads to an upregulation in both mRNA and protein expression of Cav-1. This subsequently promotes the upregulation of Slug expression, thereby enhancing the invasive and migratory capabilities of breast cancer cells. (Zielinska et al., 2018). This evidence suggests that Cav-1 may promote the invasion and migration of breast cancer by promoting the EMT process.

In addition, studies have shown that downregulation of Cav-1 in BT474 cells can also downregulate the expression of MMP-2, MMP-9 and MMP-1 and inhibit cell migration and invasion. In addition, in metastasis-associated macrophages, the deletion of Cav-1 can enhance VEGF-A/VEGFR1 activity, induce the downstream expression of MMP-9 and colony-stimulating factor-1 (CSF-1), and jointly promote angiogenesis and tumor metastatic growth. *In vitro* cell experiments showed that *Scleromitrion diffusum* can effectively inhibit the metastasis of breast cancer cells, possibly by inhibiting the expression of Cav-1 protein, thereby reducing matrix metalloproteinases (MMPs) and inhibiting its ability to invade and migrate (Yang et al., 2019). Zheng et al. (2018) also found that Antarctic Krill docosahexaenoic acid (DHA) could enhance the interaction between CD95 (Fas/APO-1) and Cav-1 in MCF-7 cells, inhibit the FAK/SRC/PI3K/AKT signaling pathway, and downregulate the expression of MMP-2, thereby inhibiting invasion and metastasis.

Rho family proteins are a group of guanosine triphosphate (GTP)-binding proteins with molecular weights ranging from 20 to 25 kDa that have GTPase activity and are also known as small G proteins, which are highly activated in a variety of malignant tumors and participate in the regulation of tumor cell morphology, extracellular matrix adhesion and cytoskeletal remodeling. Cav-1 plays an important regulatory role in tumor invasion and migration (Matsuoka and Yashiro, 2014). In inflammatory breast cancer (IBC) cells, the high expression of Cav-1 activates the Akt1 signaling pathway and phosphorylates the RhoC-GTP enzyme, thereby promoting the adhesion and migration of breast cancer cells and enhancing cell invasion (Joglekar et al., 2015). In addition, phosphorylation of the 14th tyrosine of Cav-1 can activate Rac1, another Rho family protein, by upregulating the expression of Ras-related protein 5A (Rab5). Rac1 can bind to various intracellular molecules in the form of a molecular bridge and activate various signaling pathways. For example, the PI3K/Akt/mTOR signaling pathway promotes cytoskeletal remodeling and cell invasion and migration (Diaz et al., 2014).

In addition, Cav-1 can also act on integrin, another important molecule, to promote the invasion and migration of breast cancer cells (Kozyulina et al., 2015). Integrin is a Ca^2+^ and Mg^2+^dependent heterophile cell adhesion molecule located on the cell surface that mediates the mutual recognition and adhesion between cells and between cells and the extracellular matrix. The invasion and metastasis of cells require stable adhesion between the cell pseudopods and ECM, providing a traction fulcrum for cells to migrate forward. Therefore, integrins, as transmembrane connectors connecting the extracellular matrix and intracellular actin skeleton, bind extracellular domains to extracellular ligands (such as fibridesmin and laminin), resulting in changes in the terminal configuration of intracellular domains and changes in the interactions between intracellular domains and neighboring proteins, thus activating a series of signaling cascades and connecting with the cytoskeleton. This provides anchor sites for cell migration (De Franceschi et al., 2015). The transfer-promoting protein NEDD9 can regulate the intracellular transport of integrin and the localized expression of the cell membrane through Cav-1. Vesicles expressing Cav-1 can engraft ligand-bound integrins into the cells, promote the dissociation of ligands and integrins and release integrins, thus playing an important role in cell adhesion and migration. When NEDD9 is absent, although the expression of integrin does not change, it significantly affects the adhesion of integrin on the surface of the cell membrane. Overexpression of Cav-1 phosphorylated at 14-tyrosine can restore integrin activity and promote cell invasion (Kozyulina et al., 2015).

However, Lv et al. (2016) found that macrophage migration inhibitor (MIF) can induce the phosphorylation of Cav-1 and promote the transfer of high mobility group protein B1 (HMGB1) from the cytoplasm to the ECM, thus activating TLR4 signaling and promoting breast cancer metastasis. Studies have shown that after MDA-MB231 cells escape the extracellular matrix (ECM) and enter the hemodynamic environment, low fluid shear stress can induce the upregulation of Cav-1 expression, promote the inactivation of protease caspase-8, and enhance the anti-nest loss ability of cells (Li et al., 2019). Breast cancer metastasis involves multiple steps, such as cell adhesion, movement, local invasion and migration, and circulating tumor cells (CTCs) have the ability to resist programmed apoptosis (loss of nests), so patients with metastatic breast cancer often have a poor prognosis. Gene expression profile analysis showed that the expression level of Cav-1 in bone marrow metastatic breast cancer cells was significantly higher than that in CTCs (Magbanua et al., 2018). Wang et al. (2018) found that in MDA-MB-231 cells, overexpression of Cav-1 can activate the PI3K/AKT and MEK/ERK survival signaling pathways and ITGB1-FAK signaling pathways and improve cell resistance to nest loss.

Therefore, Cav-1 may play an important role in the invasion and metastasis of breast cancer by regulating epithelial-mesenchymal transformation, extracellular matrix changes, Rho family proteins and integrin endocytosis.

#### 2.1.4 Cav-1 inhibits the formation of breast cancer stem cells

Tumor stem cells are cells in tumors that have the ability to self-renew and generate heterogeneous tumor cells. Breast cancer stem cells (BCSCs) have been found to play a key role in the proliferation, metastasis and recurrence of breast cancer (Yoon et al., 2019; Wang et al., 2020). Recently, an increasing number of studies have begun to focus on the physiological role of Cav-1 in BCSCs formation. In cloned spheres derived from breast cancer cells, the expression of Cav-1 is significantly downregulated, and inhibition of Cav-1 can upregulate indicators related to tumor stemness (CD44/CD24), promote the self-renewal ability of breast cancer stem cells, and thus promote the malignant tumor behavior of breast cancer, such as EMT, invasion and metastasis (Yoon et al., 2019). In addition, Cav-1 inhibits the self-renewal capacity and aerobic glycolysis of breast cancer stem cells through C-myc-mediated tumor metabolic reprogramming (Shi et al., 2015). The mechanism mainly shows that inhibition of Cav-1 can reduce the binding of the E3 ligase VHL to C-myc, alleviate the ubiquitination degradation of C-myc protein, and promote the accumulation of C-myc, thus leading to the orderly progress of aerobic glycolysis mediated by C-myc and providing energy support for the self-renewal ability of breast cancer stem cells. So far, some studies have indicated that Cav-1 plays an inhibitory role in BCSCs formation.

#### 2.1.5 Cav-1 mediates endocytosis of breast cancer therapeutics

Cav-1-mediated selective endocytosis is the selective transport of extracellular substances to the membranous region of the cell through the invagination vesicles formed by the cell membrane. Therefore, vesicles containing Cav-1 can carry different specific proteins to trigger the endocytosis of pits and transport them to specific organelles. This selective endocytosis plays an important role in the cell movement and migration and has a profound impact on the metabolism of therapeutic drugs (Chung et al., 2015; Chatterjee et al., 2017; Chung et al., 2018).

In the treatment of breast cancer, the uptake of certain drugs, such as albumin-paclitaxel and trastuzumab-metan conjugate (T-DM1), depends on Cav-1-mediated selective endocytosis (Chatterjee et al., 2017; Chung et al., 2018). As a new generation of paclitaxel preparations, albumin-bound paclitaxel (albumin-paclitaxel) is a first-line drug for triple-negative breast cancer chemotherapy and occupies an extremely important position in the chemotherapy of breast cancer and other malignant tumors. As a carrier of paclitaxel, albumin can bind to Cav-1 and be transported into the cell through endocytic vesicles, and paclitaxel then enters the cell along with albumin to exert its antitumor activity (Chatterjee et al., 2017). Therefore, breast cancer with high expression of Cav-1 protein is often associated with better albumin-paclitaxel treatment (Borsoi et al., 2017; Ricci et al., 2019). Cav-1-mediated endocytosis is also an important mechanism of T-DM1 endocytosis. As a second-line drug for the targeted therapy of advanced HER-2 (+) breast cancer, T-DM1 is an antibody-coupled drug consisting of trastuzumab and the anti-microtubule drug metformin. Studies have shown that Caveolae-mediated endocytosis is an important mechanism of T-DM1 resistance in HER-2 (+) breast cancer. Cav-1 is highly expressed in T-DM1-resistant HER-2 (+) breast cancer cells, and T-DM1 fuses with lysosomes intracellularly through Caveolae-mediated endocytosis, while the acidic environment in the lysosomal Cavity weakens the drug efficacy of T-DM1 (Chung et al., 2018; Sung et al., 2018). Therefore, Cav-1 may be an important target for the treatment of T-DM1 resistance.

#### 2.1.6 Cav-1 can interact with chemotherapy drugs to affect the progression of breast cancer

Salis et al. (2014a) proposed that fluvastatin can enhance cytotoxicity in MCF-7 cells by downregulating the expression of Cav-1 and serum glucocorticoid-regulated kinase 1 (SGK1). Metformin, on the other hand, enhances cytotoxicity by upregulating Cav-1 and downregulating SGK1 (Salis et al., 2014b). Overexpression of Cav-1 can increase the expression of breast cancer drug resistance protein (BCRP). Downregulation of Cav-1 can decrease the activity of ATP-binding box family G protein subfamily 2 (ABCG2) in BCRP and improve the chemotherapy sensitivity of drug-resistant breast cancer cells. Cav-1 can participate in the regulation of T-DM1 resistance by mediating cellular endocytosis (Sung et al., 2018), promoting the internalization of T-DM1 into cells and enhancing its drug toxicity and sensitivity (Chung et al., 2018). Zheng et al. (2019) proposed that overexpression of Cav-1 in MCF-7 and MDA-MB-231 cells could affect chemical sensitization by inhibiting eNOS/NO/ONOO pathway activity and oxidative damage. In addition, overexpression of Cav-1 in breast cancer can promote EGFR nuclear translocation, activate DNA-dependent protein kinase (DNAPK), induce DNA repair and enhance radiation resistance, suggesting that Cav-1 is associated with breast cancer radiotherapy (Zou et al., 2017).

#### 2.1.7 Cav-1 as an indicator of clinical prognosis

In invasive breast cancer, the expression of Cav-1 in stromal cells is associated with tumor size, histological grade, and lymph node metastasis, so Cav-1 may be a clinical diagnostic indicator of tumor prognosis.

Yeong et al. (2018) conducted histological classification and staging of 71 patients with invasive breast cancer and evaluated the vascular metastasis, lymph node metastasis, inflammatory changes in breast cancer tissue, lymph node infiltration, ER expression of estrogen receptor, p53 mutation, HER-2 expression, Ki-67 hyperplasia index, and ability of *in situ* recurrence and metastasis in tissue samples of patients. Prognostic survival time was measured, and the protein expression of Cav-1 in stromal cells and tumor cells was detected. High expression of Cav-1 in stromal cells was associated with good prognosis. Breast cancer patients with high or high expression of Cav-1 in stromal cells had higher overall survival and disease-free survival than patients with no or low expression of Cav-1. Evidence suggests that the expression level of Cav-1 in stromal cells is positively correlated with the prognosis of breast cancer patients, but the expression level of Cav-1 in tumors is not indicative of prognostic survival. In addition, the expression of Cav-1 in stromal cells was negatively correlated with the expression of estrogen receptor (ER) in breast cancer and positively correlated with tumor metastasis traits. The expression of Cav-1 in breast cancer with lymph node metastasis is significantly higher than that in breast ductal carcinoma *in situ* without lymph node metastasis, indicating that the high expression of Cav-1 is related to the invasion and metastasis of breast cancer (Eliyatkin et al., 2018)^.^

Although the presence of Cav-1 predicts a high risk of breast cancer metastasis, Cav-1 is still associated with a favorable prognosis in terms of overall survival, which may be related to the spatiotemporal specific expression of Cav-1 and the competing effects of tumor suppressors and tumor promotion. In addition, high expression of Cav-1 in breast cancer is indicative of breast cancer sensitivity to albumin-paclitaxel. In metastatic breast cancer, immunohistochemical analysis indicated that patients with high expression of Cav-1 had a significantly higher pathological response rate to albumin-paclitaxel than those with low expression of Cav-1, which provided a reference for future clinical drug guidance.

### 2.2 The relationship between Cav-2 and Cav-3 and breast cancer

Cav-2 is colocalized with Cav-1, which is closely coexpressed and has a 38% homologous sequence. Cav-2 regulates a variety of signaling pathways, which can either directly bind to Cav-1 to form hetero-oligomers or act independently of Cav-1. Studies have found that the expression of Cav-2 is upregulated in MCF-7 cells (Hnasko and Lisanti, 2003), while Shatseva et al. (2011) reported that miR-199a-3p can promote cell proliferation by inhibiting the expression of Cav-2, suggesting that Cav-2 plays a role in cancer inhibition. However, Huang et al. (2007) found that the expression of Cav-2 was downregulated after breast cancer cells received dasatinib. Savage et al. (2008) also proposed that breast cancer patients with high expression of Cav-2 had poor prognosis, and Cav-2 was negatively correlated with ER expression. Loss of Cav-2 expression can inhibit ERα phosphorylation induced by 17β-estradiol (E2), inhibit ERα transcriptional activity and activation of related signaling pathways, and thus inhibit cell proliferation. A study on IBC cells showed that Cav-2 and Cav-1 were highly expressed in IBC and were closely related to RhoC-GTP due to reduced promoter methylation (Van den Eynden et al., 2006). In addition, it has been reported that Cav-2 is correlated with Her-2 expression and triple-negative breast cancer (TNBC) (Elsheikh et al., 2008).

The Cav-3-encoding gene is located on human chromosome 3 p25, and its distribution is more limited than that of Cav-1 and Cav-2. Cav-3 is mainly expressed in vascular smooth muscle, myocardium and skeletal muscle (He et al., 2022), as well as myoepithelial cells and glial cells in the mammary gland. Mutations in the Cav-3 coding region P140L can weaken p38, AKT and endoplasmic reticulum stress signals (Stoppani et al., 2011). There are still few studies on the role of Cav-3 in breast cancer. Studies have shown that with the progression of breast cancer, the positive rate of Cav-3 in epithelial tissues decreases, while the positive rate of Cav-2 in interstitial tissues increases (Koo et al., 2011). In addition, loss of Cav-3 expression can form an anti-breast tumor microenvironment. In *in vivo* experiments, when Cav-3 is not expressed, the anti-mammary tumor formation ability of mice is significantly enhanced, while the growth of mammary tumors is inhibited and lung metastasis is significantly reduced (Sotgia et al., 2009). Therefore, Cav-2, Cav-3 and Cav-1 can also act as related regulatory factors in breast cancer progression and play dual roles in cancer inhibition and promotion.

## 3 Cavins and breast cancer

### 3.1 Relationship between Cavin-1 and breast cancer

Cavin-1 is encoded by a gene located on human chromosome 17 q21.2 and plays an important role in maintaining the structure and function of the pit. It can be attracted to the cell membrane by Cav-1 and Cav-3, bind to phosphatidylserine, cholesterol and oligomerized Caveolins, and participate in the regulation of cell membrane curvature (Liu, 2020). Cavin-1 can be released from Caveolae during insulin signaling or membrane stretch to alter transcription and protein synthesis. Cavin-1 expression is significantly downregulated in breast cancer cell lines and breast cancer tissues and is closely related to its promoter methylation. Verma et al. (2010) found that Cavin-1 was related to Cav-1. In breast cancer SK-BR-3 cells, overexpression of Cavin-1 inhibited the formation of cell membrane tubules induced by Cav-1. Other studies have shown that receptor tyrosine kinase-like orphan receptor 1 (ROR1) maintains the downstream pro-survival signaling pathway in lung adenocarcinoma by promoting the interaction of Cavin-1 and Cav-1 (Yamaguchi et al., 2016). In addition, Cavin-1 and Cav-1 can bind to insulin-like growth factor-i receptor (IGF-IR) and regulate its internalization (Hamoudane et al., 2013). Breast cancer cells contain a large amount of IGF-IR. However, the specific role of Cavin-1 and Cav-1 in regulating IGF-IR in breast cancer is still controversial and needs further study.

### 3.2 Relationship between Cavin-2 and breast cancer

The gene encoding Cavin-2 is located on human chromosome 2 q32.3 as a substrate phosphorylated by protein kinase C-PKC, affecting cell localization and substrate specificity. The Cavin-2-encoding gene can purify platelet phospholipid-binding protein (PSP68), and its expression is enhanced in serum-starved cells. It is therefore called serum deprivation response factor (Wang et al., 2019). Studies have shown that Cavin-2 expression is deficient in tumors such as breast, prostate and kidney (Nassar and Parat, 2020). Cavin-2 was found to be significantly positively correlated with the disease-free survival (DFS) and distant metastasis-free survival (DMFS) of breast cancer patients, and its loss of expression may be related to promoter methylation, while overexpression of Cavin-2 inhibited cell migration and reduced the tumor formation rate of lung metastatic tumors in NOD/SCID mice.

At the same time, the expression of the antiapoptotic protein Bcl-xL was inhibited, and apoptosis was promoted, suggesting that Cavin-2 could be used as a tumor metastasis inhibitor (Ozturk et al., 2016). Tian et al. (Tian et al., 2016) found that overexpression of Cavin-2 inhibited the proliferation and invasion ability of MDA-MB-231 cells, and loss of expression could activate the transforming growth factor TGF-β signaling pathway and induce an EMT-like phenotype in cells, suggesting that Cavin-2 could inhibit breast cancer progression by blocking the TGF-β signaling pathway. In addition, four semi-lim domain protein 1 (Fhl1) can induce Cavin-2 expression in Src protein kinase-transformed cells, independent of mitogen-activated protein kinase (MAPK) activity, and the expression of Fhl1 and Cavin-2 is significantly downregulated in breast cancer, suggesting that Cavin-2 plays a tumor suppressor role in breast cancer (Li et al., 2008).

### 3.3 Relationship between Cavin-3 and Cavin-4 and breast cancer

Cavin-3 was originally defined as a phosphatidylserine-binding protein, similar to Cavin-2, which also acts as a substrate of PKC and is induced in the serum deprivation response, and its coding gene is located in the p15.5-p15.4 region of chromosome 11 and near the D11S1323 locus. Loss of heterozygosity (LOH) in this region is often observed in sporadic breast cancer and other types of tumors. Cavin-3 is highly expressed in normal breast and lung epithelial cells, while it is absent in breast cancer, lung cancer and gastric cancer, which may be related to its promoter methylation, suggesting that Cavin-3 may play an anticancer role in breast cancer. In addition, Cavin-3 can interact with breast cancer susceptibility gene 1 (BRCA1), and its loss of expression can affect BRCA1-mediated tumor inhibition (Xu et al., 2001).

Although some studies have shown that Cavin-3 plays a role in cancer inhibition in breast cancer, the specific molecular mechanism is not fully understood. Cavin-4, as an evolutionarily conserved muscle-specific component of the Cavin complex related to the muscle-membrane Caveolae complex, encodes a gene located at q31.1 of human chromosome 9, which is also expressed at a low level in other types of cells, such as embryonic fibroblasts, and can directly interact with Cavin-2 and Cavin-3. Studies have shown that overexpression of Cavin-4 in skeletal muscle can promote myogenesis and lead to conduction disorders, while overexpression of Cavin-4 in myocardial tissue can induce cardiac dysfunction, suggesting that Cavin-4 can be used as a new potential candidate gene for muscle-associated Caveolae lesions (Bastiani et al., 2009). However, the relationship between Cavin-4 and breast cancer remains unclear.

## 4 Discussion and conclusion

As the role of Caveolae family-related proteins in the occurrence and development of breast cancer and their specific molecular mechanisms have received extensive attention, Caveolae family-related proteins are known to play important roles in the proliferation, metastasis, treatment and clinical prognostic guidance of breast cancer. Caveolae plays an important role in various physiological and pathological processes. The formation and function of the Caveolae depend on the expression and interaction of Caveolins and Cavins. However, the role of Cavins in the development of breast cancer is still controversial.

**FIGURE 2:**
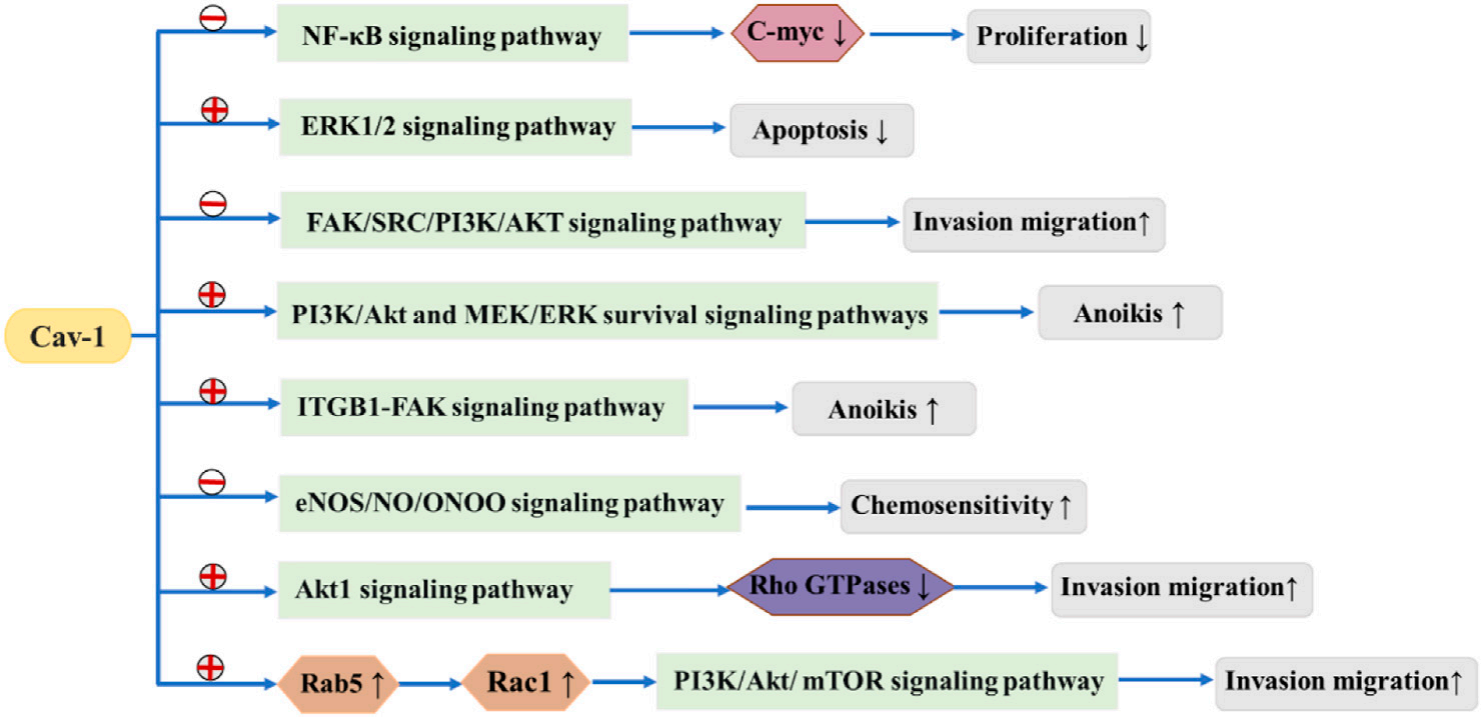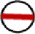 means to promote 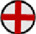
means to suppress. Cav-1 suppresses or promotes tumor signaling pathways, thereby affecting the development of breast cancer.

It has been reported that alteration of the Cav-1 gene may change the risk of breast cancer (Fard and Nafisi, 2018). It has been reported that genetic changes in Cav-1 might modify the risk for breast cancer, and Cav-1 acts as both a tumor suppressor and an oncogene and plays a key role in breast cancer tumorigenesis (Deb et al., 2014). Currently, it is believed that Cav-1 can be associated with multiple proteins and signal transduction pathways and has various regulatory effects on tumor formation, proliferation, invasion and metastasis. Caveolae act as a platform for interactions between many receptors and related signal transduction proteins, allowing Cav-1 to play an important role in regulating the balance between tumor signaling pathways (Figure 2).

Cav-1 is the focus of current research, and other Caveolae family-related proteins are less studied. Depending on the molecular classification and stage of breast cancer, Cav-2 can also play a dual role as a tumor suppressor or a cancer-promoting factor. Existing studies suggest that Cav-3 is expressed in muscle tissue and plays a dual role in tumor development, but its role in breast cancer remains to be further explored. Cavin-1, Cavin-2 and Cavin-3 are not expressed in breast cancer cells and tissues, and the specific mechanism of action of Cavin-4 in breast cancer is not very clear. The relationship between Cavin-4 and breast cancer remains to be explored in the future.

The occurrence and formation of tumors in breast cancer dare a continuous process. Although we summarized the role of Caveolae family-related proteins in various processes of breast cancer progression in this review (Table 1), the influence of Caveolae family-related proteins on breast cancer development is continuous and involves multiple processes. Although the specific mechanisms of the interactions between the Caveolae family-related proteins and various signaling molecules in different stages of tumor development and development are still unclear, we believe that a summary of the interactions between the Caveolae family-related proteins and breast cancer can lay a foundation for further research, and promote the Caveolae family-related proteins as a potential target for breast cancer diagnosis and treatment, providing a new therapeutic idea for clinical research.

**TABLE 1:** The role of Caveolae family-related proteins in various processes of breast cancer progression. Caveolae family-related proteins can act as a suppressor in breast cancer cell carcinogenic process via suppressing breast cancer cell proliferation, autophagy, invasion and migration and promoting apoptosis. Caveolae family-related proteins can also act as a promoter in breast cancer cell carcinogenic process via promoting breast cancer cell proliferation, autophagy, invasion, migration and metastasis, and suppressing apoptosis and anoikis.

Caveolae family-related proteins	Signaling cascades	Progress stages
Cav-1↑	SDF-1,EGF,FSP-1↑	Proliferation↑
Cav-1↑	Glucolysis↓	Proliferation↓
Cav-1↑	Autolysosome↓	Proliferation↓
Cav-1↓	ERα↑	Proliferation↑
Cav-1↓	BKCa↑	Proliferation↑
Cav-1↑	p21,p27, cyclin B1↑ cyclin D2↓	Proliferation↓
Cav-1↑	EGFR signaling ↑	Proliferation↑
Cav-1↑	HSP90, HIF-1α, VEGF↑	Proliferation↑
Cav-2↓	miR-199a-3p → Cav-2↓	Proliferation↓
Cav-2↓	Dasatinib → Cav-2↓	Proliferation↓
Cav-2↑	ERα↓	Proliferation↓
Cav-3↓	Anti-tumor microenvironment↑	Proliferation↓ Metastasis↓
Cav-1↑	DTX→Cav-1↑→ apoptotic pathways	Apoptosis↑
Cav-1↓	V-ATPase↑→ autophagosome↑	Apoptosis↓
Cav-1↑	cyclin D1,β-catenin↑→ autophagosome↑	Apoptosis↓
Cav-1↓	MβCD→Cav-1↓→ survivin,Bcl-2,Bax↑	Apoptosis↑
Cavin-2↑	Bcl-xL↓	Apoptosis↑
Cav-1↑	Slug↑	Invasion, migration↑
Cav-1↓	MMP-2,MMP-9,MMP-1↓	Invasion, migration↓
Cav-1↓	VEGF-A/VEGFR1↑→MMP-9,CSF-1↑	Metastasis↑
Cav-1↓	Scleromitrion diffusum → Cav-1↓→ MMPs↓	Invasion, migration↓
Cav-1↓	DHA→Cav1-Fas↑→ MMP-2↓	Invasion, migration↓
Cav-1↑	RhoC - GTPase phosphorylation↑	Invasion, migration↑
Cav-1↑	Rab5↑→ Rac1↑	Invasion, migration↑
Cav-1↑	integrin↑	Invasion, migration↑
Cav-1↑	caspase-8↓→ anoikis↓	Invasion, migration↑
Cavin-2↓	TGF-β signaling pathway↑	Proliferation↓ Invasion↓
